# A refined rodent model of anorexia nervosa: Simulating state‐specific effects of caloric restriction and weight restoration

**DOI:** 10.14814/phy2.16092

**Published:** 2024-06-21

**Authors:** Megan E. Rosa‐Caldwell, Lauren Breithaupt, Ursula B. Kaiser, Eliza Garland, Sheridyn Pinkham, Madisyn Hancock, Sophie L. Jalkut, Seward B. Rutkove

**Affiliations:** ^1^ Department of Neurology Beth Israel Deaconess Medical Center, Harvard Medical School Boston Massachusetts USA; ^2^ Department of Health Human Performance and Recreation, Exercise Science Research Center University of Arkansas Fayetteville Arkansas USA; ^3^ Eating Disorders Clinical and Research Program, Department of Psychiatry Harvard Medical School, Massachusetts General Hospital Boston Massachusetts USA; ^4^ Mass General Brigham Multidisciplinary Eating Disorders Research Collaborative, Mass General Brigham Boston Massachusetts USA; ^5^ Division of Endocrinology Diabetes and Hypertension, Brigham and Women's Hospital, Harvard Medical School Boston Massachusetts USA

**Keywords:** anorexia nervosa, bone mineral density, estrous cycle, pQCT, rats, rodent model

## Abstract

Current rodent models of anorexia nervosa (AN) often have accelerated weight loss that often do not allow for investigation of physiological ramifications of prolonged low weight status characteristic of AN. The purpose of this project was to refine a rodent model of AN to extend the duration of low weight status and allow for investigation of recovery. Eight‐week‐old female Sprague Dawley rats underwent 50%–60% food restriction for 30 days. Rats were group‐housed except during feeding, where AN rats were individually housed and given up to 2 h to consume food. Control (CON) rats were allowed to consume food ad libitum. To simulate recovery, a separate cohort of animals underwent the same food restriction protocol for 30 days, then rats (AN‐R) were allowed to consume food ad libitum to facilitate weight recovery for an additional 30 days. AN‐R rats were compared to age matched controls (CON‐R). AN rats lost ~15% bodyweight and were ~30% lighter than CON. Compared to CON, AN rats had ~35% lower fat content, ~18% lower bone mineral density, ~22% smaller plantaris muscle mass and ~52% smaller ovaries. Upon reintroduction of food, AN‐R rats achieved comparable bodyweights to CON‐R rats after ~10 days. However, after 30 days of recovery, AN‐R rats still had ~14% lower bone mineral density and ~11% smaller plantaris mass and ~21% smaller ovaries. This refinement of rodent AN results in physiological side effects of AN without reaching excessive weight loss requiring euthanasia. Moreover, some physiological consequences of simulated AN are not resolved with weight restoration.

## 1 INTRODUCTION

Anorexia nervosa (AN) and its associated health complications continue to remain a challenge for clinicians. AN (either prior AN or current AN) affects millions of individuals in the United States, with poor recovery rates (Deloitte Access Economics, 2020; Eddy et al., 2017; Micali et al., 2017; Smink et al., 2013). Correspondingly, quality of life and overall health are reduced in individuals with this condition. Few treatment options currently exist that effectively mitigate AN's health consequences. This may in part be due to relatively limited research on the consequent physiological alterations that occur during active AN or with weight recovery. Historically, animal models have facilitated a more thorough understanding of biological mechanisms for multiple disease pathologies. In fact, animal models (typically rodent) have facilitated many medical breakthroughs for various diseases. However, recapitulating AN in rodents has multiple challenges.

One of the most common methods to induce AN in rodents is the activity‐based anorexia model (ABA), whereby animals are given restricted access to food and free access to a running wheel (Scherma et al., 2019). With this model, rodents often reach ~20%–25% weight loss within ~7 days, which may necessitate animal euthanasia to mitigate suffering per many Institutional Animal Care and Use Committees' (IACUCs) regulations (Frintrop et al., 2018; Giles et al., 2016; Gilman et al., 2019; Perez‐Leighton et al., 2014; Scherma et al., 2019). While this model clearly elicits the food restriction and hyperactivity of AN, this model has several shortcomings. First, this model is highly accelerated and does not allow for the sustained low weight status seen in many individuals with AN (Austin et al., 2021; Breithaupt et al., 2022; Eddy et al., 2017). Second, while over‐exercising is reported in 31%–80% of individuals with AN (Dalle Grave et al., 2008; Fietz et al., 2014), exercise itself results in a milieu of physiological alterations that may have both independent and interactive effects with the low energy status induced by low caloric intake (Hawley et al., 2018, 2021).Moreover, rodents typically run 6–10 km a day on average (Chen et al., 2017; Frintrop et al., 2018; Giles et al., 2016; Scherma et al., 2019), given the size of the animals and total exercise volume, this exercise volume may not be physiologically realistic to compare to humans. Finally, the ABA model necessitates singly housing rodents. Isolation results in stress for rodents (Manouze et al., 2019); correspondingly, many IACUCs prefer group housing rodents whenever possible to maintain animal welfare. While this single housing is necessary to directly monitor running wheel volume, singly housing rodents results in additional stressors that may interact with the model itself. For example, rodents utilize huddling to maintain body temperature (Hankenson et al., 2018). When rodents are singly housed, they do not have the ability to huddle and thus must utilize other means to maintain body temperature. This is often accomplished by increased activity to generate body heat (e.g., running) (Hankenson et al., 2018). Coupled with reduced food intake, this additional physical activity generates a vicious feedforward cycle and elicits the highly accelerated weight loss seen in the ABA model. Other models of AN such as stress‐induced food restriction or genetic models also have various short comings, such as only transient decreases in total food intake (not long‐term), rodents dying before they reach 3 weeks of age (prior to weaning), or being very logistically challenging to induce (IAK, 2019; Madra & Zeltser, 2016; Wang, 2002).

Given these model shortcomings, we set out to refine a restricted‐feeding model of AN that would include group housing to attenuate weight loss in rats. The purpose of this paper is to describe our refinement to previous animal models of AN that allows for investigation of long‐term food restriction as well as weight restoration. This model recapitulates key physiological features of AN, such as sustained low weight, low fat mass, altered estrous cycle (rodent equivalent of menstrual cycle), and low bone mineral density relative to healthy control rats. We believe this refined model can be used to investigate the physiological ramifications of low energy consumption.

## 2 METHODS

### 2.1 Overall design

All protocols were approved by the Beth Israel Deaconess Medical Center (BIDMC) Institutional Animal Care and Use Committee (Animal Use Protocol # 009–2022). All animals were kept on a 12:12 h dark/light cycle in a facility kept at ~23°C. For this initial model, we only used female rats because AN disproportionally affect females compared to males in the general population by a ratio of ~4:1 (Galmiche et al., 2019; van Eeden et al., 2021). During all interventions, rats had access to enrichment (e.g., toys) in their home cages as well as access to water. Female SAS Sprague Dawley rats (Charles River, Bar Harbor, ME, Strain code: 400) were obtained at 6 weeks of age (*n* = 11/group). After 48 h of acclimatization to facilities, estrous cycle was monitored daily (described below). At ~8 weeks of age (adolescence) rats began simulated AN food restriction (described below). Rats completed 30 days of stimulated AN, which roughly translates to ~2.5–3 years in humans with rats of this age (Andreollo et al., 2012; Quinn, 2005). Baseline bodyweights were not different between groups (CON: 174.7 ± 10.9 grams, AN: 172.2 ± 14.4 grams, *p* = 0.63).

In a separate cohort, rats underwent simulated AN procedure for 30 days. Afterwards, rodents were provided food ad libitum to facilitate recovery. Rats underwent recovery interventions for 30 days (AN‐R) and compared to age‐matched healthy controls (CON‐R). Baseline bodyweights were also not different between groups in this cohort (CON‐R: 175.9 ± 9.4 grams, AN‐R: 181.9 ± 12.6 grams, *p* = 0.22).

For euthanasia, all rats were fasted ~20 h. The next day, rats were given a standard amount of food (~3 grams) ~90 min prior to euthanasia to normalize feeding status across groups. Rats consumed food in ~30 min. Approximately 60 min after food consumption, rats were anesthetized with 2.5% isoflurane and tissues (ovaries, skeletal muscle, liver, and heart) collected and weighed. Heart was collected last and resulted in rat euthanasia. These procedures are aligned with 2020 American Veterinary Medical Association Guidelines on Euthanasia guidelines. The time between feeding and euthanasia did not differ between groups in either cohort. (CON: 108.4 ± 19.0 min v AN: 126.4 ± 34.7 min, *p* = 0.16 and CON‐R: 93.8 ± 4.0 min v. AN‐R: 95.4 ± 3.6 min, *p* = 0.37).

### 2.2 Simulated anorexia nervosa food restriction and recovery

Baseline food consumption of all rats was monitored for ~3 days prior to food restriction. On the first day of food restriction, AN rats were removed from group housing and placed in individual cages. Rats were then provided ~50%–60% less food than baseline consumption. We opted for 50%–60% food restriction because patients with AN commonly consume 30%–80% fewer calories than they require (Golden et al., 2013; Marzola et al., 2013; Peebles et al., 2017); therefore, we anticipated ~50% food restriction would appropriately mirror the clinical condition. Rats were allowed up to 2 h to consume food, after which they were placed back in group housing (AN rats housed together). If rats consumed food in <2 h, then rats were returned to group housing. After ~5–7 days on protocol, most rats consumed food in <30 min. Rats remained in group housing until feeding the following day, when AN rats were again placed individually in cages for feeding and provided ~50%–60% less food than baseline consumption. CON and CON‐R rats were allowed ad libitum access to food throughout all interventions. AN rats were fed between 8:00 and10:00 am every day (during light cycle). Rats were provided standard laboratory rodent chow containing 26.6% protein, 16.5% fat, and 56.8% carbohydrate (LabDiet Formulab Diet, catalog no. 5008, irradiated). Rats' bodyweight was monitored daily; if an animal had >20% bodyweight loss sustained over a 48 h period, the animal was euthanized, per BIDMC animal welfare policy. During this protocol, no rats required euthanasia due to excessive bodyweight loss.

### 2.3 Assessment of estrous cycles

As altered menstrual cycle is a common occurrence in women with AN (Puckett et al., 2021), we assessed the rodent corollary, the estrous cycle. Estrous cycles were assessed by vaginal lavage with collection and imaging of vaginal cells with 0.01% crystal violet staining, as previously described (Rosa‐Caldwell et al., 2021; Rosa‐Caldwell, Mortreux, et al., 2023a). Vaginal lavages were collected prior to feeding in AN rats and ~ 8:00 am in CON rats. One investigator (MER) classified vaginal lavage slides as proestrus, estrus, metestrus, or diestrus. The investigator was blinded to animal groups while classifying vaginal lavage slides. Data were quantified as percentage of time spent in either metestrus or diestrus (low estrogen phases).

### 2.4 pQCT protocol

Peripheral quantitative computed tomography (pQCT) was completed as previously described (Stratee, XCT Research SA+, Pforzheim, Germany) (Rosa‐Caldwell, Mortreux, et al., 2023a). Rats were anesthetized with ~2.5% isoflurane, rats were then placed on a custom platform with continuous isoflurane anesthesia via a nose cone. pQCT images were obtained 40 mm distal from the tibial plateau with a voxel size of 0.10 mm and Ct speed 10 mm/s. The entire scan lasted ~10 min. Three images were obtained sequentially and the average value of the three images was calculated. Images were quantified for total fat area and bone mineral density using density thresholds and filters provided by the manufacturer.

### 2.5 Novel object recognition

As a metric of cognitive function, novel object recognition (NOR) was be performed as previously described (Antunes & Biala, 2012; Mathiasen & DiCamillo, 2010). Forty‐eight hours prior to euthanasia, a rat was placed on one end of a 12in × 12in cage with a plastic divider. On the other side of the divider, two objects of similar size, color and shape were placed. The divider was then removed, and the rat was allowed to explore the two objects for 5 min (familiarization phase). After 5 min, the rat was removed and placed back in its home cage. The next day, the rat was placed in the cage again and one of the two objects was replaced with a novel object of approximately equal size but different color and shape of the “familiar object.” Rats were allowed to explore the cage and the novel object for 3 min (testing phase). Testing was completed at a standard time of day (~10 am), in a low noise standardized room with dim lighting. Objects were disinfected inbetween animals with Clidox disinfectant solution. During testing, the location of the familiar object (right or left side of cage) was switched between each rat. Variables of interest were time taken to recognize novel object, percentage of time spent investigating novel object v. familiar object (calculated as time investigating novel object/time spent investigating novel and familiar object), percentage of interactions with novel object v. familiar object (calculated as frequency of novel object interactions/sum of interactions for novel and familiar object) (Paulukat et al., 2016), total time spent investigating either object and total number of interactions with either object. Investigation of object included: nose within ~1 cm of object, turning/moving the object, and/or touching the object. All NOR trials were recorded and analyzed by at least two researchers, if any outcome variable differed by >2 seconds/interactions, then a third reviewer analyzed the video. Values were averaged across all reviewers. Correlation between reviewers across all NOR outcomes ranged from 0.75 to 0.89, indicating overall good reliability across reviewers.

### 2.6 Statistical analysis

All data were assessed for normality and outliers prior to analysis. If an outlier resulted in significant skew or was greater than two standard deviations from the mean, that data point was removed. Food consumption and bodyweight were analyzed with repeated measures analysis of variance (ANOVA, factors of time and group). A Tukey post‐hoc was used to control for multiple comparisons. Estrous cycle was analyzed with custom statistical code to evaluate the differences between groups at each time point with a Holm's post‐hoc used to control for Type 1 error rate. We have previously used versions of this code in previous manuscripts to increase statistical power for specific comparisons of interest (Rosa‐Caldwell, Mortreux, et al., 2023a, 2023b). Other data were analyzed with *t*‐test. For variables that could be influenced by baseline bodyweight (fat area, bone mineral density, tissue weights), we used a covariate of baseline bodyweight. Significance was denoted at *p* < 0.05. All analysis was completed in SAS statistical software (SAS Institute, Cary, NC, United States). All raw data, statistical code, and statistical outputs for skew analysis and overall analysis are available on our Open Science Framework page (https://doi.org/10.17605/OSF.IO/R6S45).

## 3 RESULTS

### 3.1 Food restriction resulted in loss of bodyweight in AN rats, without reaching required euthanasia due to excessive weight loss

**FIGURE 1:**
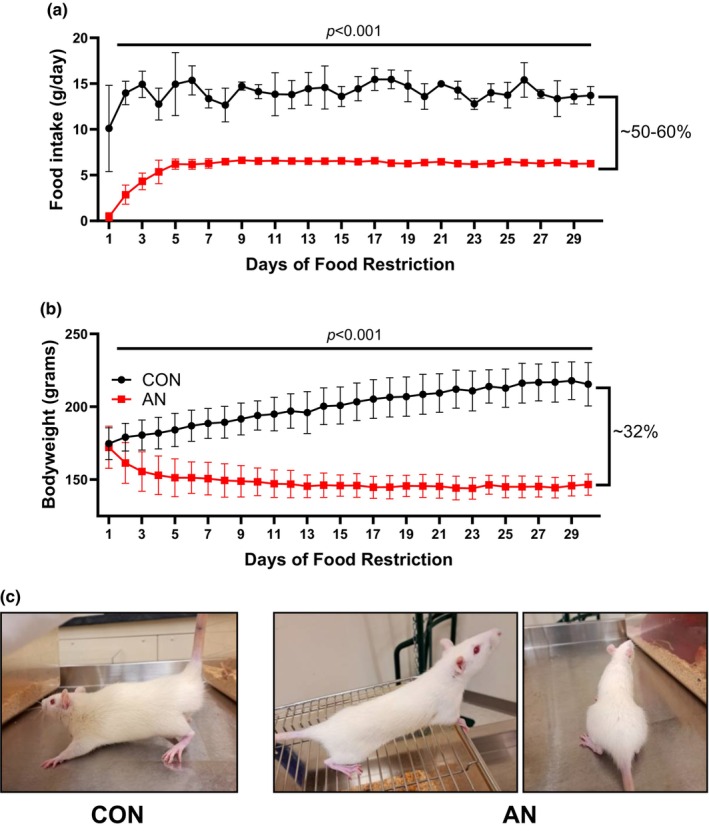
Food intake and bodyweight data for simulated model of AN in rats. (a) Daily food intake during 30‐day food restriction intervention. (b) Daily bodyweight for 30‐day food restriction intervention. (c) Representative images of CON and AN rats after 30 day interventions. CON = control rats, AN = simulated anorexia nervosa rats. Data are plotted as mean ± STD.

**FIGURE 2:**
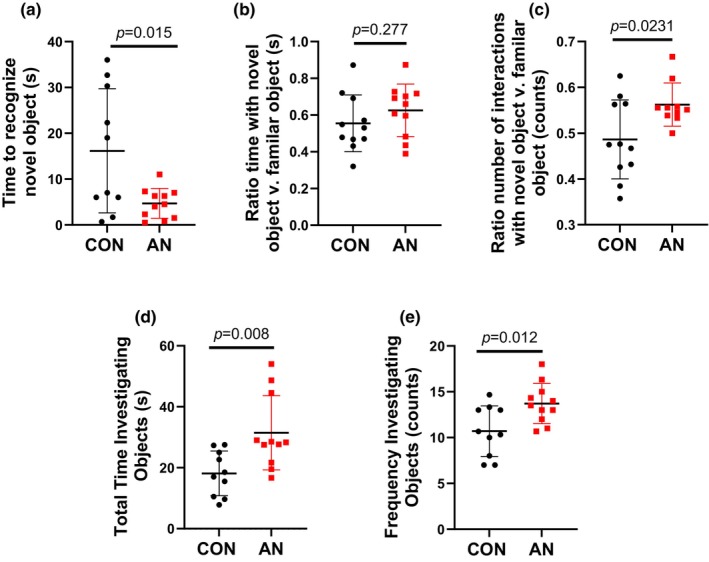
Novel object recognition parameters after simulated AN. (a) Time to recognize novel object during NOR testing. (b) Ratio of time spent investigating novel object relative to familiar object. (c) Ratio of number of interactions with novel object compared to familiar object. (d) Total time spent investigating objects (both novel and familiar). (e) Total number of interactions with objects (both novel and familiar). CON = control rats, AN = simulated anorexia nervosa rats. Data are plotted as mean ± STD.

With initiation of food restriction, AN rats ate significantly less food compared to CON. Within the first few days, AN rats did not consume all food provided, but consumed all food provided by Day 5 (Figure 1a). Overall, AN rats consumed ~55% less food compared to CON (Figure 1a). Correspondingly, AN rats also lost ~14.5% bodyweight (Figure 1b). With the loss of bodyweight in AN rats combined with weight gain in CON rats, there was ~32% difference in bodyweight between AN and CON rats (Figure 1b). No animals required premature euthanasia due to excessive weight loss (>20%), poor body condition score (<2), or behavior indicative of excessive stress/pain (lack of water consumption, lethargy, etc). Representative images of AN and CON rats are depicted in Figure 2c.

### 3.2 AN rats did not display behavior characteristic of altered memory on during novel object recognition (NOR)

AN rats overall found the novel object ~15 s faster compared to CON (Figure 2a), did not investigate the novel object more than familiar object compared to CON (Figure 2b), and had ~15% more interactions with the novel object compared to CON (Figure 2c). Additionally, AN rats had ~70% greater time with objects (novel and familiar) and ~34% greater number of interactions with objects (novel and familiar) compared to CON (Figure 2d,e).

### 3.3 AN rats displayed physiological features characteristic of clinical AN

**FIGURE 3:**
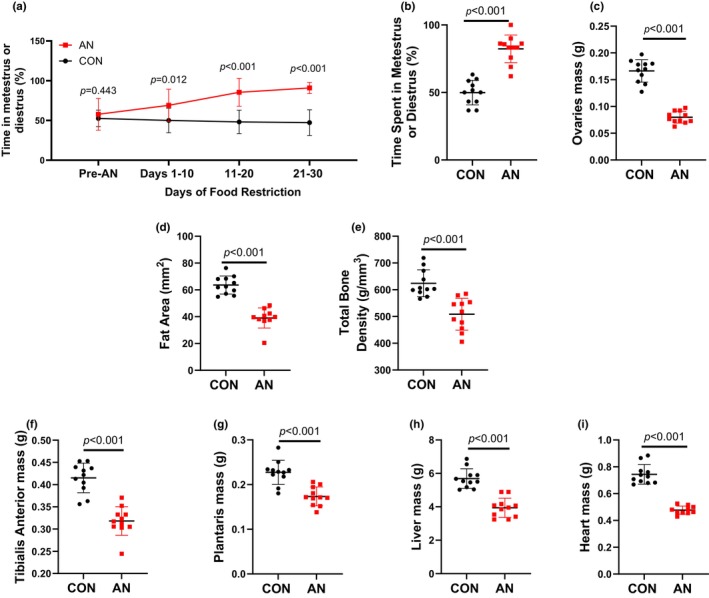
Physiological data during and after simulated AN. (a) Percentage of days spent in either metestrus or diestrus prior to AN and across 10 day intervals during simulated AN. (b) Total percentage of days spent in either metestrus or diestrus during simulated AN. (c) Ovary mass after simulated AN. (d) Fat area measured by pQCT in the lower leg after simulated AN. (e) Bone mineral density measured by pQCT in the tibia 40 mm distal from tibial plateau after simulated AN. (f) Tibialis anterior mass after simulated AN. (g) Plantaris mass after simulated AN. (h) Liver mass after simulated AN. (i) Heart mass after simulated AN. CON = control rats, AN = simulated anorexia nervosa rats. Data are plotted as mean ± STD.

Within 10 days of food restriction, AN rats spent more time in low estrogen states metestrus and diestrus (Figure 3a). This pattern remained throughout the duration of food restriction (Figure 3a). Overall, AN rats spent more time in metestrus or diestrus compared to CON (82.2% vs. 49.9%, Figure 3b). Correspondingly, AN rats had ~52% smaller ovaries compared to CON rats (Figure 3c). AN rats had ~38% lower fat area and ~18% bone mineral density as measured by pQCT (Figure 3d,e). Finally, AN rats had ~22% lower tibialis anterior, ~22% lower plantaris, ~30% lower liver, and 26% lower heart mass compared to CON (Figure 3f–i).

### 3.4 With reintroduction of food, AN‐R rats quickly regained weight; however, many physiological features of AN did not fully normalize after weight gain

**FIGURE 4:**
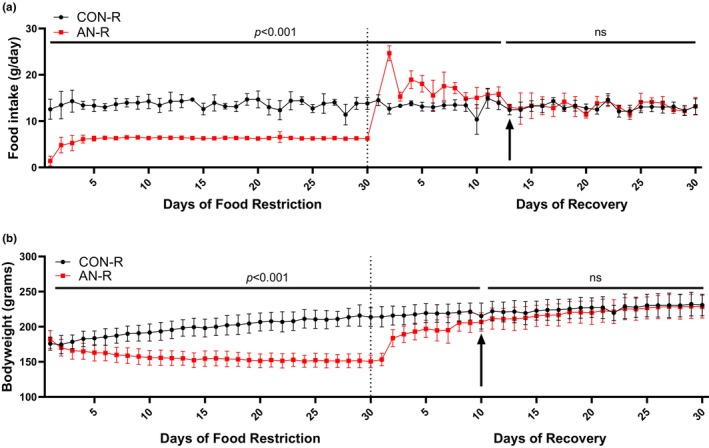
Food intake and bodyweight data after simulated AN for 30 days followed by 30 days of ad libitum food intake recovery. (a) Daily food intake during 30‐day food restriction intervention and 30 day recovery intervention. (b) Daily bodyweight for 30‐day food restriction intervention and 30 day recovery intervention. Dotted lines indicate the start of recovery. Arrows indicate where AN‐R and CON‐R food intake or bodyweight were not different between groups. CON‐R = aged matched healthy control rats. AN‐R = simulated anorexia nervosa rats followed by recovery. Data are plotted as mean ± STD.

**FIGURE 5:**
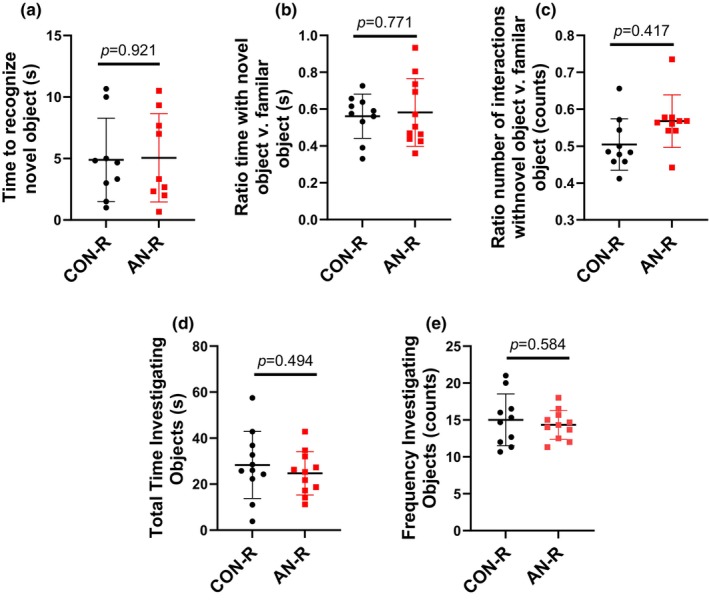
Novel object recognition parameters after simulated AN and 30‐day weight recovery interventions. (a) Time to recognize novel object during NOR testing. (b) Ratio of time spent investigating novel object relative to familiar object. (c) Ratio of number of interactions with novel object compared to familiar object. (d) Total time spent investigating objects (both novel and familiar). (e) Total number of interactions with objects (both novel and familiar). CON‐R = aged matched healthy control rats. AN‐R = simulated anorexia nervosa rats followed by recovery. Data are plotted as mean ± STD.

**FIGURE 6:**
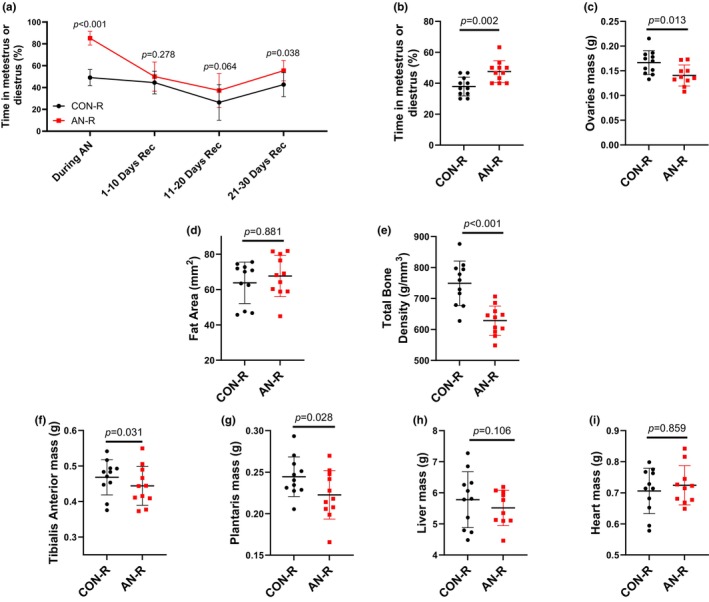
Physiological data after simulated AN and 30‐day weight recovery. (a) Percentage of days spent in either metestrus or diestrus during simulated AN and across 10‐day intervals during recovery intervention. (b) Total percentage of days spent in either metestrus or diestrus during recovery intervention. (c) Ovary mass after simulated AN and recovery. (d) Fat area measured by pQCT in the lower leg after simulated AN and recovery. (e) Bone mineral density measured by pQCT in the tibia 40 mm distal from tibial plateau after simulated AN and recovery. (f) Tibialis anterior mass after simulated AN and recovery. (g) Plantaris mass after simulated AN and recovery. (h) Liver mass after simulated AN and recovery. (i) Heart mass after simulated AN and recovery. CON‐R = aged matched healthy control rats. AN‐R = simulated anorexia nervosa rats followed by recovery. Data are plotted as mean ± STD.

Upon ad libitum access to food, AN‐R rats consumed more food than CON‐R animals. AN‐R rats quickly regained bodyweight; by 10–13 days of recovery, food consumption and bodyweight were similar between CON‐R and AN‐R animals (Figure 4a,b). After weight gain interventions, no metrics of NOR were different between CON‐R and AN‐R (Figure 5a–e). With refeeding, AN‐R animals spent less time in low estrogen phases metestrus and diestrus (Figure 6a). However, across the entire recovery period, AN‐R spent greater time in metestrus or diestrus compared to CON‐R (Figure 6b). AN‐R rats also had ~21% smaller ovaries compared to CON‐R (Figure 6c). Fat area was not different between CON‐R and AN‐R rats (~65mm^2^, Figure 6d); however, bone density was still lower in AN‐R compared to CON‐R (14% lower, Figure 6e). Muscle mass of both the tibialis anterior and plantaris were lower in AN‐R compared to CON‐R (~9% and ~11% respectively, Figure 6f,g). Liver mass and heart mass were not different in AN‐R compared to CON‐R (~5.6 grams and ~0.71 grams respectively, Figure 6h,i).

## 4 DISCUSSION

Animal models are vital to understand the physiological ramifications of the low energy state characteristic of AN. We believe our refinement of classic restricted feeding paradigms improves on previous methods. Specifically, we extend duration of food restriction without reaching required euthanasia endpoints, while still achieving key physiological features of AN. Additionally, we reduce overall stress to the rats by allowing group housing and social interactions. Finally, this model allows for the investigation of long‐term health complications of AN following weight recovery, which is often very difficult to investigate in human participants.

In the current model, we recapitulated key features of AN, including low bodyweight, oligomenorrhea/amenorrhea (as measured through estrous cycle), low body fat, low bone mineral density, and lower tissue weights (Puckett et al., 2021; Rosa‐Caldwell, Eddy, et al., 2023) (Figures 1 and 3). These features suggest that this model is sufficient to recapitulate physiological features of AN. Of note, because we use young rats (to simulate adolescence/young adulthood), we have a combination of weight loss and diminution of normal weight gain. These combined stressors result in ~30%–32% difference in weight between AN and CON rats. To compare this difference to humans, if a human control group had an average BMI of 23 kg/m^2^, a 30%–32% lower bodyweight would be equivalent to a BMI of 15.6–16.1 kg/m^2^, well within the diagnostic criteria for moderate or severe AN according to DSM‐5 standards (Allen et al., 2013; Toppino et al., 2022). Moreover, this relative difference in bodyweights is more representative of clinical AN. For example, in ABA models, AN rodents are often ~35%–45% smaller compared to control animals (Scharner & Stengel, 2021), whereas in clinical studies the difference between healthy controls and those with AN typically ranges from 20% to 30% (Keeler et al., 2024; Kerruish et al., 2002; Lyall et al., 2024). Importantly, none of our animals required euthanasia due to excessive weight loss, low body condition score, or dramatic changes in behavior. Therefore, this model attenuates animal stress and attrition, while still eliciting physiological symptoms of AN.

We believe the greatest advantage of this refined restricted feeding model is the extended duration of simulated AN, which we believe is driven by group housing the rats and avoiding wheel running. Rodents use huddling and nesting in order to maintain body heat, especially during energetic stress (Hankenson et al., 2018). Group housing facilitates huddling, thereby reducing caloric expenditure to maintain body heat and reducing overall stress. Moreover, removing the running wheel allows for longer protocol durations. When rodents are not able to sufficiently maintain body heat via huddling, they will typically increase movement (Hankenson et al., 2018). Correspondingly, rats given a running wheel will exercise to generate body heat. This running wheel behavior (6–10 km in dark cycle on average (Chen et al., 2017; Frintrop et al., 2018; Giles et al., 2016; Scherma et al., 2019)) results in aggressive weight loss within a very short timeframe (~7 days). We should also note that rats may be running more due to isolation‐induced stress as some studies find rodents will run more at thermoneutrality compared to colder temperatures (McKie et al., 2019). Regardless of the precise mechanism for running behavior, the increased activity in ABA models results in accelerated timelines that do not allow for the investigation of physiological changes that may be occurring prior to lethal weight loss or recapitulate the prolonged low weight status (>5 years) seen in many clinical studies of AN (Kaufmann et al., 2021; Toppino et al., 2022). Our model includes food restriction for 30 days, which to our knowledge is longer than many other rodent models of AN, and considering our rat's overall condition, we believe the duration of food restriction could be extended even farther. Rats reach the low weight status by ~10–15 days of restriction and then remain at a low weight status for the remainder of the intervention. Therefore, we can characterize temporal changes that occur during the development of the low weight status. Finally, using this restricted feeding and then re‐feeding paradigm, we note that AN‐R rats gain weight equal to CON‐R animals within ~10 days; however, despite weight restoration rats still display altered physiology (greater overall time spent in low estrogen phases, smaller ovaries, lower bone mineral density, lower muscle mass) compared to control rats (Figures 4 and 6). These results suggest physiological consequences of AN may be longer lasting than previously thought, especially in the context of AN occurring during times of growth such as adolescence/teenage years.

We did not note any differences in cognition as measured through NOR. In fact, contrary to our original hypothesis, AN rats found the novel object faster, spent more time with the novel object, and overall interacted with the objects more than CON rats (Figure 2). This finding is contrary to previous research implementing NOR in rodents with simulated AN (Boersma et al., 2016; Paulukat et al., 2016). Importantly, these earlier studies used ABA models of AN, one where animals were low weight status (Paulukat et al., 2016) the other when rats were weight recovered (Boersma et al., 2016). We postulate these findings from previous research may be a hitherto underappreciated interaction between weight loss and isolation. Prior research has noted that isolation (e.g., single housing) itself is sufficient to result in differences in a NOR task (Bianchi et al., 2006; Manouze et al., 2019; McLean et al., 2010). Correspondingly, it is possible singly housing rodents in previous studies may have contributed to differences noted in NOR. Additionally, considering ABA was utilized in previous studies (Boersma et al., 2016; Paulukat et al., 2016), it is also possible the greater weight loss (~25% vs. ~15%) contributed to differences in NOR, suggesting degree of weight loss may be an important contributor to cognitive ramifications of AN, both during low weight status (Paulukat et al., 2016) and after weight recovery (Boersma et al., 2016).

Hyperactivity is a common symptom of AN, both clinically and in rodents (Dalle Grave et al., 2008; Scharner & Stengel, 2021). In rodent models of AN, this is typically measured and induced via running wheels. While not a direct measure of movement, we did anecdotally observe AN animals moved more within their cages and were more active compared to their CON counterparts, but unfortunately, we were unable to directly quantify this movement. This general observation of hyperactivity is supported by our NOR data, where AN rats had more overall interactions and movement within the cage (Figure 2). These data suggest we induced the hyperactivity characteristic of AN. This is supported by the NOR data in our AN‐R rats demonstrating no differences in NOR across all parameters (Figure 5). While more objective measures of activity would confirm these findings, our current data implies hyperactivity in our rodent model of AN.

Using this refined AN model and refeeding, we find some of the physiological implications of AN are not resolved with weight recovery. This is despite AN‐R rats achieving a similar bodyweight to CON‐R rats and sustaining that weight for >15 days (Figure 4). For example, AN‐R rodents overall spent more time in low estrogen phases throughout the 30‐day recovery period and ovary mass was still smaller compared to CON‐R. As estrogens are considered critical for optimal function across multiple organ systems, including reproductive, musculoskeletal, and cardiovascular (Collins et al., 2019; Gersh et al., 2024; Manolagas et al., 2013), this lack of recovery in estrous cycle could contribute to alterations in overall health. Moreover, we also note bone mineral density and muscle mass also do not recover with weight restoration. Skeletal health (or lack thereof) is a key modulator of fracture risk, and muscle mass is a key regulator of skeletal health through Wolfe Law mechanisms (Frost, 2003; Lang et al., 2009). Therefore, long‐term alterations to skeletal muscle mass have a direct influence on fracture risk (Sornay‐Rendu et al., 2017). These findings of prolonged physiological consequences of AN have been noted in the clinical literature (Mueller et al., 2015; Rosa‐Caldwell, Eddy, et al., 2023); however, these long‐term investigations in clinical populations are notoriously difficult to perform. Therefore, this refined model of AN for rodents provides an opportunity to more directly evaluate long‐term implications of AN and ideally generate interventions to mitigate these physiological consequences.

There are some limitations to the model which we must acknowledge. First, we are not able to directly compare this model to an ABA cohort. In part, we are not able to match ABA animals on age/duration of food restriction given the accelerated timeline of ABA (~7 days before ~25% weight loss) (Frintrop et al., 2018; Giles et al., 2016; Gilman et al., 2019; Perez‐Leighton et al., 2014; Scherma et al., 2019). It would be ideal if we could directly compare the two models; however, that would entail either starting ABA in older rats (so they could be age matched to our model) or starting protocols at the same age and comparing differently aged animals, either of which would complicate overall comparison between the two models and data interpretability. However, because we did not directly compare our model to ABA we are limited in our direct conclusions about our model compared to ABA. Additionally, in our current model, rats were fed between 8 and 10 am, which is slightly outside of their normal light:dark cycle (7 am: 7 pm). Therefore, there is a possibility for some circadian alterations. We attempted to mitigate this shortcoming as much as possible by feeding animals early in the morning. Additionally, our CON animals remained group housed throughout the entire protocol and were not placed into separate cages for feeding as was the case for the AN animals. While we feel it is unlikely this <2 h/day difference in housing makes a dramatic difference in physiological outcomes, we cannot exclude the possibility that this slight difference in housing may influence results. We also only tested this model in female rodents, due to AN disproportionally affecting females (~4–8× more prevalent in females compared to males) (Galmiche et al., 2019; van Eeden et al., 2021); however, in the future this model should be validated in male rodents. Finally, this model does not replicate the volitional food restriction, seen with human AN. So, we are unable to disentangle potential biological mechanisms driving low food intake.

In conclusion, our refinement of classic restricted feeding models of AN provides the necessary phenotypic characteristics of AN, while overall improving animal welfare compared to previous models. We believe this rodent model will allow for a more thorough understanding of the physiological underpinnings of AN and facilitate future therapeutic treatments.

## ETHICS STATEMENT

All protocols were approved by the Beth Israel Deaconess Medical Center (BIDMC) Institutional Animal Care and Use Committee (Animal Use Protocol # 009–2022).

## IRB STATEMENT

Not applicable for this manuscript.

## Data Availability

All data presented in this manuscript are available through this manuscript's Open Science Framework Page (https://doi.org/10.17605/OSF.IO/R6S45).

## References

[phy216092-bib-0001] Allen, K. L. , Byrne, S. M. , Oddy, W. H. , & Crosby, R. D. (2013). DSM‐IV‐TR and DSM‐5 eating disorders in adolescents: Prevalence, stability, and psychosocial correlates in a population‐based sample of male and female adolescents. Journal of Abnormal Psychology, 122, 720–732. 24016012 10.1037/a0034004

[phy216092-bib-0002] Andreollo, N. A. , Santos, E. F. , Araújo, M. R. , & Lopes, L. R. (2012). Rat's age versus human's age: What is the relationship? Arquivos Brasileiros de Cirurgia Digestiva: ABCD = Brazilian Archives of Digestive Surgery, 25(1), 49–51. 22569979 10.1590/s0102-67202012000100011

[phy216092-bib-0003] Antunes, M. , & Biala, G. (2012). The novel object recognition memory: neurobiology, test procedure, and its modifications. 10.1007/s10339-011-0430-z PMC3332351 22160349

[phy216092-bib-0004] Austin, A. , Flynn, M. , Richards, K. , Hodsoll, J. , Duarte, T. A. , Robinson, P. , Kelly, J. , & Schmidt, U. (2021). Duration of untreated eating disorder and relationship to outcomes: A systematic review of the literature. European Eating Disorders Review, 29(3), 329–345. 32578311 10.1002/erv.2745

[phy216092-bib-0005] Bianchi, M. , Fone, K. F. , Azmi, N. , Heidbreder, C. A. , Hagan, J. J. , & Marsden, C. A. (2006). Isolation rearing induces recognition memory deficits accompanied by cytoskeletal alterations in rat hippocampus. The European Journal of Neuroscience, 24, 2894–2902. 17116162 10.1111/j.1460-9568.2006.05170.x

[phy216092-bib-0006] Boersma, G. J. , Treesukosol, Y. , Cordner, Z. A. , Kastelein, A. , Choi, P. , Moran, T. H. , & Tamashiro, K. L. (2016). Exposure to activity‐based anorexia impairs contextual learning in weight‐restored rats without affecting spatial learning, taste, anxiety, or dietary‐fat preference. The International Journal of Eating Disorders, 49, 167–179. 26711541 10.1002/eat.22489 PMC4777973

[phy216092-bib-0007] Breithaupt, L. , Kahn, D. L. , Slattery, M. , Plessow, F. , Mancuso, C. , Izquierdo, A. , Dreier, M. J. , Becker, K. , Franko, D. L. , Thomas, J. J. , Holsen, L. , Lawson, E. A. , Misra, M. , & Eddy, K. T. (2022). Eighteen‐month course and outcome of adolescent restrictive eating disorders: Persistence, crossover, and recovery. Journal of Clinical Child and Adolescent Psychology: The Official Journal for the Society of Clinical Child and Adolescent Psychology, American Psychological Association, Division, 53, 51–725. 10.1080/15374416.2022.2034634 PMC9444807 35476589

[phy216092-bib-0008] Chen, Y. W. , Actor‐Engel, H. , Sherpa, A. D. , Klingensmith, L. , Chowdhury, T. G. , & Aoki, C. (2017). NR2A‐ and NR2B‐NMDA receptors and drebrin within postsynaptic spines of the hippocampus correlate with hunger‐evoked exercise. Brain Structure & Function, 222(5), 2271–2294. 27915379 10.1007/s00429-016-1341-7 PMC5764086

[phy216092-bib-0009] Collins, B. C. , Laakkonen, E. K. , & Lowe, D. A. (2019). Aging of the musculoskeletal system: How the loss of estrogen impacts muscle strength. Bone, 123, 137–144. 30930293 10.1016/j.bone.2019.03.033 PMC6491229

[phy216092-bib-0010] Dalle Grave, R. , Calugi, S. , & Marchesini, G. (2008). Compulsive exercise to control shape or weight in eating disorders: Prevalence, associated features, and treatment outcome. Comprehensive Psychiatry, 49(4), 346–352. 18555054 10.1016/j.comppsych.2007.12.007

[phy216092-bib-0011] Deloitte Access Economics . (2020). The social and economic cost of eating disorders in The United States of America: A report for the strategic training initiative for the prevention of eating disorders and the academy for eating disorders.

[phy216092-bib-0012] Eddy, K. T. , Tabri, N. , Thomas, J. J. , Murray, H. B. , Keshaviah, A. , Hastings, E. , Edkins, K. , Krishna, M. , Herzog, D. B. , Keel, P. K. , & Franko, D. L. (2017). Recovery from anorexia nervosa and bulimia nervosa at 22‐year follow‐up. The Journal of Clinical Psychiatry, 78, 184–189. 28002660 10.4088/JCP.15m10393 PMC7883487

[phy216092-bib-0013] Fietz, M. , Touyz, S. , & Hay, P. (2014). A risk profile of compulsive exercise in adolescents with an eating disorder: A systematic review.

[phy216092-bib-0014] Frintrop, L. , Trinh, S. , Liesbrock, J. , Paulukat, L. , Kas, M. J. , Tolba, R. , Konrad, K. , Herpertz‐Dahlmann, B. , Beyer, C. , & Seitz, J. (2018). Establishment of a chronic activity‐based anorexia rat model. Journal of Neuroscience Methods, 293, 191–198. 28970163 10.1016/j.jneumeth.2017.09.018

[phy216092-bib-0015] Frost, H. M. A. (2003). Update of bone physiology and Wolff's law for clinicians. The Angle Orthodontist, 74, 2004. 10.1043/0003-3219(2004)074<0003:AUOBPA>2.0.CO;2 15038485

[phy216092-bib-0016] Galmiche, M. , Déchelotte, P. , Lambert, G. , & Tavolacci, M. P. (2019). Prevalence of eating disorders over the 2000‐2018 period: A systematic literature review. The American Journal of Clinical Nutrition, 109, 1402–1413. 31051507 10.1093/ajcn/nqy342

[phy216092-bib-0017] Gersh, F. , O'Keefe, J. H. , Elagizi, A. , Lavie, C. J. , & Laukkanen, J. A. (2024). Estrogen and cardiovascular disease. Progress in Cardiovascular Diseases, S0033‐0620(24)00015‐X. 10.1016/j.pcad.2024.01.015 38272338

[phy216092-bib-0018] Giles, E. D. , Hagman, J. , Pan, Z. , MacLean, P. S. , & Higgins, J. A. (2016). Weight restoration on a high carbohydrate refeeding diet promotes rapid weight regain and hepatic lipid accumulation in female anorexic rats. Nutrition & Metabolism, 13, 18. 26937246 10.1186/s12986-016-0077-y PMC4773993

[phy216092-bib-0019] Gilman, T. L. , Owens, W. A. , George, C. M. , Metzel, L. , Vitela, M. , Ferreira, L. , Bowman, M. A. , Gould, G. G. , Toney, G. M. , & Daws, L. C. (2019). Age‐ and sex‐specific plasticity in dopamine transporter function revealed by food restriction and exercise in a rat activity‐based anorexia paradigm. The Journal of Pharmacology and Experimental Therapeutics, 371, 268–277. 31481515 10.1124/jpet.119.260794 PMC6795746

[phy216092-bib-0020] Golden, N. H. , Keane‐Miller, C. , Sainani, K. L. , & Kapphahn, C. J. (2013). Higher caloric intake in hospitalized adolescents with anorexia nervosa is associated with reduced length of stay and no increased rate of refeeding syndrome. The Journal of Adolescent Health, 53, 573–578. 23830088 10.1016/j.jadohealth.2013.05.014

[phy216092-bib-0021] Hankenson, F. C. , Marx, J. O. , Gordon, C. J. , & David, J. M. (2018). Effects of rodent thermoregulation on animal models in the research environment. Comparative Medicine, 68(6), 425–438. 30458902 10.30802/AALAS-CM-18-000049 PMC6310197

[phy216092-bib-0022] Hawley, J. A. , Joyner, M. J. , & Green, D. J. (2021). Mimicking exercise: What matters most and where to next? The Journal of Physiology, 599, 791–802. 31749163 10.1113/JP278761 PMC7891316

[phy216092-bib-0023] Hawley, J. A. , Lundby, C. , Cotter, J. D. , & Burke, L. M. (2018). Maximizing cellular adaptation to endurance exercise in skeletal muscle. Cell Metabolism, 27, 962–976. 29719234 10.1016/j.cmet.2018.04.014

[phy216092-bib-0024] Nilsson, s. (2019). The anx/anx mouse—A valuable resource in anorexia nervosa research. Frontiers in Neuroscience, 13, 59. 30804742 10.3389/fnins.2019.00059 PMC6370726

[phy216092-bib-0025] Kaufmann, L. K. , Moergeli, H. , & Milos, G. F. (2021). Lifetime weight characteristics of adult inpatients with severe anorexia nervosa: Maximal lifetime BMI predicts treatment outcome. Frontiers in Psychiatry, 12, 682952. 34335330 10.3389/fpsyt.2021.682952 PMC8319499

[phy216092-bib-0026] Keeler, J. L. , Bahnsen, K. , Wronski, M. L. , Bernardoni, F. , Tam, F. , Arold, D. , King, J. A. , Kolb, T. , Poitz, D. M. , Roessner, V. , Treasure, J. , Himmerich, H. , & Ehrlich, S. (2024). Longitudinal changes in brain‐derived neurotrophic factor (BDNF) but not cytokines contribute to hippocampal recovery in anorexia nervosa above increases in body mass index. Psychological Medicine, 1–12. 10.1017/S0033291724000394 PMC11413355 38450444

[phy216092-bib-0027] Kerruish, K. P. , O'Connor, J. , Humphries, I. R. , Kohn, M. R. , Clarke, S. D. , Briody, J. N. , Thomson, E. J. , Wright, K. A. , Gaskin, K. J. , & Baur, L. A. (2002). Body composition in adolescents with anorexia nervosa. The American Journal of Clinical Nutrition, 75, 31–37. 11756057 10.1093/ajcn/75.1.31

[phy216092-bib-0028] Lang, D. H. , Conroy, D. E. , Lionikas, A. , Mack, H. A. , Larsson, L. , Vogler, G. P. , Vandenbergh, D. J. , Blizard, D. A. , McClearn, G. E. , & Sharkey, N. A. (2009). Bone, muscle, and physical activity: Structural equation modeling of relationships and genetic influence with age. Journal of Bone and Mineral Research: The Official Journal of the American Society for Bone and Mineral Research, 24(9), 1608–1617. 19419307 10.1359/JBMR.090418 PMC2730930

[phy216092-bib-0029] Lyall, A. E. , Breithaupt, L. , Ji, C. , Haidar, A. , Kotler, E. , Becker, K. R. , Plessow, F. , Slattery, M. , Thomas, J. J. , Holsen, L. M. , Misra, M. , Eddy, K. T. , & Lawson, E. A. (2024). Lower region‐specific gray matter volume in females with atypical anorexia nervosa and anorexia nervosa. The International Journal of Eating Disorders, 57, 951–966. 38366701 10.1002/eat.24168 PMC11018478

[phy216092-bib-0030] Madra, M. , & Zeltser, L. M. (2016). BDNF‐Val66Met variant and adolescent stress interact to promote susceptibility to anorexic behavior in mice. Translational Psychiatry, 6(4), e776. 27045846 10.1038/tp.2016.35 PMC4872394

[phy216092-bib-0031] Manolagas, S. C. , O'Brien, C. A. , & Almeida, M. (2013). The role of estrogen and androgen receptors in bone health and disease. Nature Reviews. Endocrinology, 9, 699–712. 10.1038/nrendo.2013.179 PMC3971652 24042328

[phy216092-bib-0032] Manouze, H. , Ghestem, A. , Poillerat, V. , Bennis, M. , Ba‐M'hamed, S. , Benoliel, J. J. , Becker, C. , & Bernard, C. (2019). Effects of single cage housing on stress, cognitive, and seizure parameters in the rat and mouse pilocarpine models of epilepsy. eNeuro, 6(4), ENEURO.0179‐18.2019. 10.1523/ENEURO.0179-18.2019 PMC6709207 31331937

[phy216092-bib-0033] Marzola, E. , Nasser, J. A. , Hashim, S. A. , Shih Pan, B. , & Kaye, W. H. (2013). Nutritional rehabilitation in anorexia nervosa: Review of the literature and implications for treatment. BMC Psychiatry, 13, 290. 24200367 10.1186/1471-244X-13-290 PMC3829207

[phy216092-bib-0034] Mathiasen, J. R. , & DiCamillo, A. (2010). Novel object recognition in the rat: A facile assay for cognitive function. Current Protocols in Pharmacology, Chapter 5:Unit 5.59. 10.1002/0471141755.ph0559s49 22294372

[phy216092-bib-0035] McKie, G. L. , Medak, K. D. , Knuth, C. M. , Shamshoum, H. , Townsend, L. K. , Peppler, W. T. , & Wright, D. C. (2019). Housing temperature affects the acute and chronic metabolic adaptations to exercise in mice. The Journal of Physiology, 597, 4581–4600. 31297830 10.1113/JP278221

[phy216092-bib-0036] McLean, S. , Grayson, B. , Harris, M. , Protheroe, C. , Woolley, M. , & Neill, J. (2010). Isolation rearing impairs novel object recognition and attentional set shifting performance in female rats. Journal of Psychopharmacology, 24, 57–63. 18635708 10.1177/0269881108093842

[phy216092-bib-0037] Micali, N. , Martini, M. G. , Thomas, J. J. , Eddy, K. T. , Kothari, R. , Russell, E. , Bulik, C. M. , & Treasure, J. (2017). Lifetime and 12‐month prevalence of eating disorders amongst women in mid‐life: A population‐based study of diagnoses and risk factors. BMC Medicine, 15, 12. 28095833 10.1186/s12916-016-0766-4 PMC5240354

[phy216092-bib-0038] Mueller, S. M. , Immoos, M. , Anliker, E. , Drobnjak, S. , Boutellier, U. , & Toigo, M. (2015). Reduced bone strength and muscle force in women 27 years after anorexia nervosa. The Journal of Clinical Endocrinology and Metabolism, 100, 2927–2933. 26086327 10.1210/jc.2015-1011

[phy216092-bib-0039] Paulukat, L. , Frintrop, L. , Liesbrock, J. , Heussen, N. , Johann, S. , Exner, C. , Kas, M. J. , Tolba, R. , Neulen, J. , Konrad, K. , Herpertz‐Dahlmann, B. , Beyer, C. , & Seitz, J. (2016). Memory impairment is associated with the loss of regular oestrous cycle and plasma oestradiol levels in an activity‐based anorexia animal model. The World Journal of Biological Psychiatry: The Official Journal of the World Federation of Societies of Biological Psychiatry, 17(4), 274–284. 27160428 10.3109/15622975.2016.1173725

[phy216092-bib-0040] Peebles, R. , Lesser, A. , Park, C. C. , Heckert, K. , Timko, C. A. , Lantzouni, E. , Liebman, R. , & Weaver, L. (2017). Outcomes of an inpatient medical nutritional rehabilitation protocol in children and adolescents with eating disorders. Journal of Eating Disorders, 5, 7. 28265411 10.1186/s40337-017-0134-6 PMC5331684

[phy216092-bib-0041] Perez‐Leighton, C. E. , Grace, M. , Billington, C. J. , & Kotz, C. M. (2014). Role of spontaneous physical activity in prediction of susceptibility to activity based anorexia in male and female rats. Physiology & Behavior, 135, 104–111. 24912135 10.1016/j.physbeh.2014.06.001 PMC4426852

[phy216092-bib-0042] Puckett, L. , Grayeb, D. , Khatri, V. , Cass, K. , & Mehler, P. (2021). A comprehensive review of complications and new findings associated with anorexia nervosa. Journal of Clinical Medicine, 10(12), 2555. 34207744 10.3390/jcm10122555 PMC8226688

[phy216092-bib-0043] Quinn, R. (2005). Comparing rat's to human's age: How old is my rat in people years? Nutrition (Burbank, Los Angeles County, Calif), 21(6), 775–777. 15925305 10.1016/j.nut.2005.04.002

[phy216092-bib-0045] Rosa‐Caldwell, M. E. , Eddy, K. T. , Rutkove, S. B. , & Breithaupt, L. (2023). Anorexia nervosa and muscle health: A systematic review of our current understanding and future recommendations for study. The International Journal of Eating Disorders, 56(3), 483–500. 36529682 10.1002/eat.23878

[phy216092-bib-0046] Rosa‐Caldwell, M. E. , Mortreux, M. , Kaiser, U. B. , Sung, D. M. , Bouxsein, M. L. , Dunlap, K. R. , Greene, N. P. , & Rutkove, S. B. (2021). The estrous cycle and skeletal muscle atrophy: Investigations in rodent models of muscle loss. Experimental Physiology, 106, 2472–2488. 34569104 10.1113/EP089962 PMC8639792

[phy216092-bib-0047] Rosa‐Caldwell, M. E. , Mortreux, M. , Wadhwa, A. , Kaiser, U. B. , Sung, D. M. , Bouxsein, M. L. , & Rutkove, S. B. (2023a). Influence of gonadectomy on muscle health in micro‐ and partial‐gravity environments in rats. Journal of Applied Physiology (Bethesda, MD: 1985), 134(6), 1438–1449. 37102698 10.1152/japplphysiol.00023.2023 PMC10228673

[phy216092-bib-0048] Rosa‐Caldwell, M. E. , Mortreux, M. , Wadhwa, A. , Kaiser, U. B. , Sung, D. M. , Bouxsein, M. L. , & Rutkove, S. B. (2023b). Sex differences in muscle health in simulated micro‐ and partial‐gravity environments in rats. Sports Medicine and Health Science, 5(4), 319–328. 38314043 10.1016/j.smhs.2023.09.002 PMC10831389

[phy216092-bib-0049] Scharner, S. , & Stengel, A. (2021). Animal models for anorexia nervosa‐a systematic review. Frontiers in Human Neuroscience, 14, 596381. 33551774 10.3389/fnhum.2020.596381 PMC7854692

[phy216092-bib-0050] Scherma, M. , Collu, R. , Satta, V. , Giunti, E. , & Fadda, P. (2019). Animal models of eating disorders. Methods in Molecular Biology (Clifton, NJ), 2011, 297–314. 10.1007/978-1-4939-9554-7_17 31273706

[phy216092-bib-0051] Smink, F. R. , van Hoeken, D. , & Hoek, H. W. (2013). Epidemiology, course, and outcome of eating disorders. Current Opinion in Psychiatry, 26, 543–548. 24060914 10.1097/YCO.0b013e328365a24f

[phy216092-bib-0052] Sornay‐Rendu, E. , Duboeuf, F. , Boutroy, S. , & Chapurlat, R. D. (2017). Muscle mass is associated with incident fracture in postmenopausal women: The OFELY study. Bone, 94, 108–113. 27989649 10.1016/j.bone.2016.10.024

[phy216092-bib-0053] Toppino, F. , Longo, P. , Martini, M. , Abbate‐Daga, G. , & Marzola, E. (2022). Body mass index specifiers in anorexia nervosa: Anything below the "extreme"? Journal of Clinical Medicine, 11(3), 542. 35159994 10.3390/jcm11030542 PMC8837073

[phy216092-bib-0054] van Eeden, A. E. , van Hoeken, D. , & Hoek, H. W. (2021). Incidence, prevalence and mortality of anorexia nervosa and bulimia nervosa. Current Opinion in Psychiatry, 34, 515–524. 34419970 10.1097/YCO.0000000000000739 PMC8500372

[phy216092-bib-0055] Wang, S. W. (2002). Effects of restraint stress and serotonin on macronutrient selection: A rat model of stress‐induced anorexia. Eating and Weight Disorders: EWD, 7, 23–31. 11930983 10.1007/BF03354426

